# blastjs: a BLAST+ wrapper for Node.js

**DOI:** 10.1186/s13104-016-1938-1

**Published:** 2016-02-27

**Authors:** Martin Page, Dan MacLean, Christian Schudoma

**Affiliations:** Bioinformatics Group, The Sainsbury Laboratory, Norwich Research Park, Norwich, NR4 7UH UK; Triticeae Genomics Group, The Genome Analysis Centre, Norwich Research Park, Norwich, NR4 7UH UK

**Keywords:** Sequence analysis, Database search, Annotation, Web services, Application development, JavaScript

## Abstract

**Background:**

To cope with the ever-increasing amount of sequence data generated in the field of genomics, the demand for efficient and fast database searches that drive functional and structural annotation in both large- and small-scale genome projects is on the rise. The tools of the BLAST+ suite are the most widely employed bioinformatic method for these database searches. Recent trends in bioinformatics application development show an increasing number of JavaScript apps that are based on modern frameworks such as Node.js. Until now, there is no way of using database searches with the BLAST+ suite from a Node.js codebase.

**Results:**

We developed blastjs, a Node.js library that wraps the search tools of the BLAST+ suite and thus allows to easily add significant functionality to any Node.js-based application.

**Conclusion:**

blastjs is a library that allows the incorporation of BLAST+ functionality into bioinformatics applications based on JavaScript and Node.js. The library was designed to be as user-friendly as possible and therefore requires only a minimal amount of code in the client application. The library is freely available under the MIT license at https://github.com/teammaclean/blastjs.

## Findings

### Background

The algorithms of the BLAST-family [1–3] are the most widely employed methods for efficiently searching biological databases. Biologists employ BLAST as a first choice when performing sequence analysis because of the widely available public interfaces, in particular NCBI BLAST (http://blast.ncbi.nlm.nih.gov) [4]. Developers of genomics and bioinformatics resources therefore seek solutions to provide BLAST functionality to their user community. Various customisable server-side versions of BLAST that present web interfaces have been developed, including wwwblast (http://www.ncbi.nlm.nih.gov/staff/tao/URLAPI/wwwblast) [3], mimicking the public interface at the NCBI-BLAST website [4], ViroBLAST [5], blastGraphic (http://gmod.org/) [6], SequenceServer (http://sequenceserver.com)[7], and galaxy_blast [8]. Downstream tools such as BLASTPLOT [9] facilitate the assessment of BLAST results via output processing and/or visualisation.

Rich web-application development is greatly facilitated by taking advantage of the power of modern web browsers and JavaScript. Running applications within a web browser eliminates the need for complex and expensive commercial graphical user interface (GUI) libraries. It thus revolutionises the development of GUI applications with an emphasis on user experience by allowing for the easy creation of user-friendly, intuitive, and truly platform-independent software solutions.

With the current amount of massive data sets being generated in the life sciences, the efficient integration of front- and backend, e.g. for data handling and presentation/visualisation, is essential. Recent developments have given rise to JavaScript tools and frameworks such as BioJS (http://biojs.net) [10] and bionode (http://www.bionode.io).

With JavaScript, efficient server-side data processing can now be done by Node.js, an open source JavaScript framework powered by Google’s V8 JavaScript engine. It enables developers to create fast and scalable web applications while using a common code base for front- and backend development, thus eliminating redundancies on the code level and avoiding other language-based bottlenecks.

As a community-driven project and recent web standard, Node.js is very well maintained and under constant development. Its active developer base produces highly useful modules such as persistent client–server connections (socket.io) or mobile application development (meteorJS).

We present blastjs, a JavaScript library based on Node.js that provides access to the search tools of the BLAST+ suite and enables the integration of BLAST-based sequence similarity searches into web applications built with Node.js.

### Implementation

We designed blastjs for simplicity and user-friendliness. To provide access to the individual BLAST+ applications, we created individual functions to easily access blastn, blastp, blastx, tblastn, tblastx, and makeblastdb (cf. Table 1) from a locally installed BLAST+ csuite. The wrapper functions were created to be asynchronous via callbacks, thus guaranteeing a smoother user experience, as the application does not lock up while a search is running. blastjs can automatically download and install the latest BLAST+ version from the NCBI website. However, as differences between versions of BLAST+ could lead to differences in scientific results, this feature has to be executed by the user in order to keep control over the used BLAST+ version.

**Table 1:** BLAST+ applications wrapped by blastjs

blastjs function	BLAST+ App	Query	Database
Search applications			
blast.blastN	blastn	Nucleotide	nucleotide
blast.blastP	blastp	protein	protein
blast.blastX	blastx	translated nucleotide	protein
blast.tblastN	tblastn	protein	translated nucleotide
blast.tblastX	tblastx	translated nucleotide	translated nucleotide
Database applications			
blast.makeDB	makeblastdb	Generate new BLAST+ database	

The wrapped BLAST+ applications can be accessed via an application programming interface (API). blastjs utilises the Node.js child_process.exec() API function (https://nodejs.org/api/child_process.html) to interact with the BLAST+ applications. The call to child_process.exec() spawns a new system process (supervised by the Node.js framework), which executes the specified BLAST+ program and communicates with it via stdin, stdout, and stderr.

As the overhead for Node.js is small, using BLAST+ via blastjs requires similar system resources to running BLAST+ applications from the command line. Hence, the number of BLAST+ processes is only limited by the underlying hardware.

**Fig. 1:**
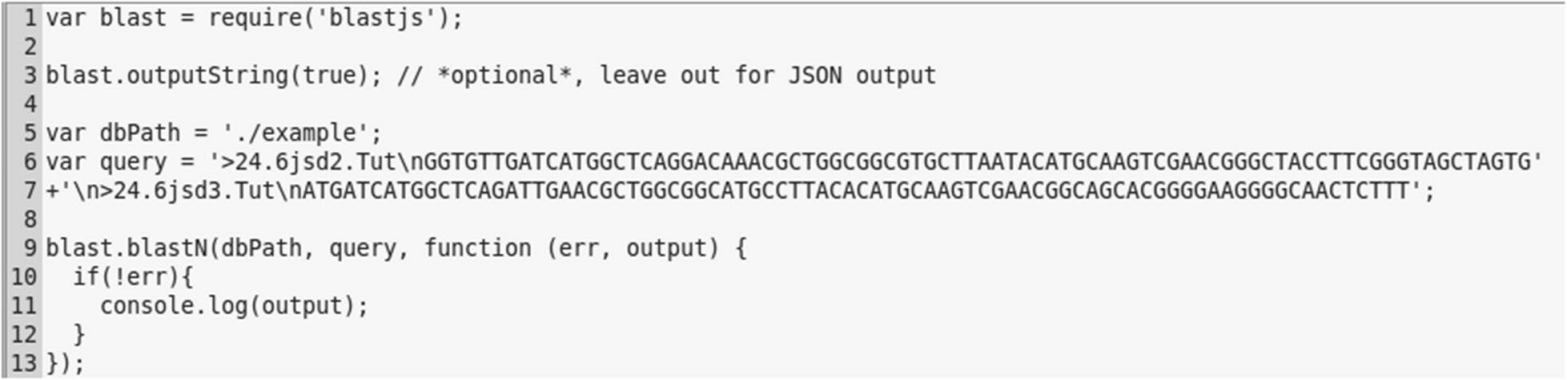
Example of a blastn search with blastjs. Integrating blastjs into a JavaScript application requires only a minimal amount of code. The example shows how to obtain BLAST+ output as text or alternatively as JSON (*line* 3) for a multi-sequence query (*lines* 6, 7)

BLAST+ searches via blastjs are easily executed and require only a small amount of code in the client application (Fig. 1). The API functions for the BLAST+ applications take as input a string containing one or more query sequence(s), and a BLAST+ database. For instance, the blastn application can be accessed using the blast.blastN() function. Advanced parameters can be directly passed to the function as JavaScript objects. For backwards compatibility with older versions of BLAST+, results are obtained in XML format (outfmt 5) from the BLAST+ applications and then converted into JavaScript Object Notation (JSON) objects. The blastjs library can thus be easily included into any Node.js application.

Furthermore, to facilitate downstream processing of the results, the generated JSON can be converted to JavaScript objects via ‘JSON.parse()’. A sample sequence file as well as a sample database are included in the example_data folder of the blastjs Github repository.

In order to be able to quickly generate custom databases, especially by an end user, we have wrapped the BLAST+ makeblastdb tool, which can be used via an API function as well.

### Results and discussion

With the current trend in application development moving towards solutions that can be run in a modern web browser, the demand for code solutions based on modern and performant JavaScript frameworks such as Node.js is on the rise.

For bioinformatics application development, however, this shift to a new environment implies an inherent lack of pre-existing bioinformatics functionality, as the corresponding code libraries are traditionally built for use in languages such as Perl or Python and thus have to be ported to be usable in JavaScript.

**Fig. 2:**
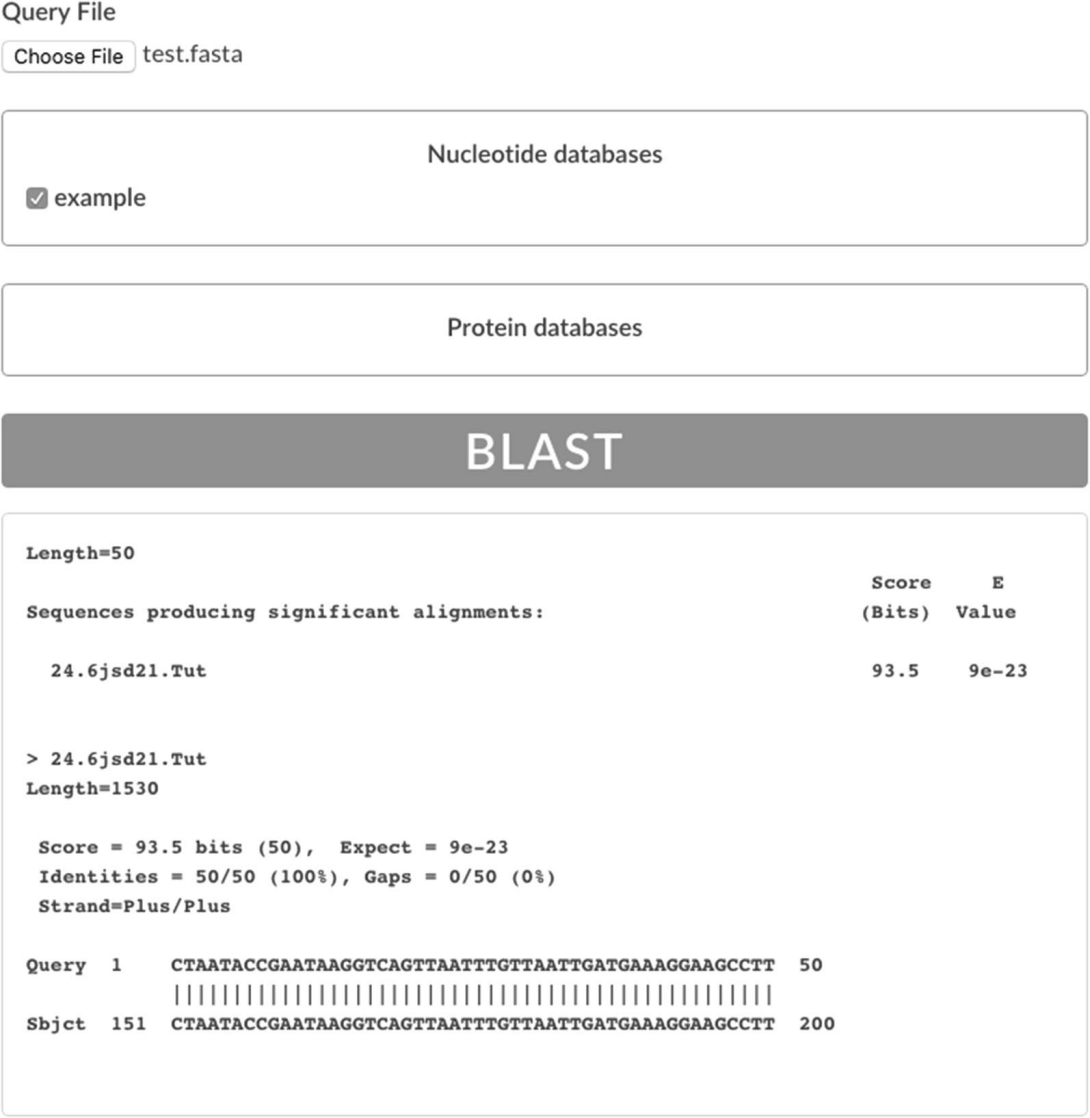
Example graphical user interface of a simple Node.js application using blastjs. As blastjs can be embedded into any Node.js codebase, user interfaces are highly customisable

To our knowledge, blastjs is the first solution for including BLAST+ database searches into Node.js-based web applications. The library profits from Node.js’s high performance in terms of speed and efficiency and can be easily integrated into any Node.js project. As blastjs simply provides an API to interact with the BLAST+ suite, GUI development is highly customisable. For an example user interface see Fig. 2.

### Conclusion

Our blastjs library provides an easy and effortless way for bioinformatics application developers of adding significant functionality (in form of the five most commonly used BLAST+ applications) to bioinformatics applications based on Node.js. We employ blastjs for in-house applications such as the genomics data resource geefu.io (http://geefu.io). As an Open Source project under the MIT license, blastjs is freely available for users to fix issues or request or add new features. Potential extensions for blastjs could be the implementation of wrappers for other BLAST-applications such as MegaBLAST or PSI-BLAST.

## Availability and requirements

- *Project name* blastjs
- *Project home page*https://github.com/teammaclean/blastjs
- *Operating system*(*s*) platform-independent
- *Programming language* JavaScript
- *Other requirements* Node.js
- *License* MIT
- *Any restrictions to use by non-academics* None
